# Iatrogenic subclavian artery rupture: A unique case report balloon occlusion

**DOI:** 10.1016/j.ijscr.2023.108508

**Published:** 2023-07-14

**Authors:** Shivam Khatri, Steven Epstein, Simon Kashfi, Parind Oza

**Affiliations:** a CUNY School of Medicine, New York, NY 10031, United States of America; b Interventional and Vascular Radiologist, Department of Radiology, St. Barnabas Hospital, Bronx, NY 10457, United States of America; c Division of Internal Medicine, Department of Medicine, Donald and Barbara Zucker School of Medicine at Hofstra/Northwell, Northwell Health, Hempstead, United States of America; d Division of Vascular Surgery, Department of Surgery, St. Barnabas Hospital, Bronx, NY 10457, United States of America

**Keywords:** Balloon occlusion, Subclavian artery, Diameter, Central venous catheter, Stent graft

## Abstract

**Introduction:**

Inadvertent subclavian artery puncture during attempted central venous catheterization can be devastating. Percutaneous stent grafting, closure devices and conventional surgery have been described to effect repair. Balloon occlusion has also been described and often recommended. Numerous publications advise use of balloon expanded to no less than the diameter of the punctured artery.

**Case presentation:**

We describe the case of a 21 year-old male whose right subclavian artery was inadvertently punctured after central-line removal. Our balloon when expanded was purposely slightly smaller than the inner arterial diameter. Balloon occlusion nevertheless alone sufficed to effect closure and repair.

**Clinical discussion:**

When the expanded balloon surpasses the diameter of the subclavian artery, it is expected that blood flow to the upper extremity will be cut off. Presently, there is a lack of definitive data concerning the maximum duration for balloon inflation that could lead to upper extremity ischemia. However, in this particular case, there was a temporary reduction in vertebral artery flow, while the flow in the carotid and axillosubclavian arteries remained rapid and uninterrupted. To our understanding, only one other case documents the use of a balloon shorter than the vessel diameter.

**Conclusion:**

In specific scenarios, it is advisable to consider the use of smaller balloon diameters to effectively stop extravasation while ensuring adequate perfusion to the brain and hand. While alternative approaches such as open repair, and stent graft procedure have been reported for repairing subclavian artery rupture, balloon tamponade provides interventional radiologists with a distinct technical advantage.

## Highlights

• Numerous publications advise use of balloon expanded to no less than the diameter of the punctured artery.
• Balloon occlusion offers a less invasive alternative to surgery or stent-graft placement in the treatment of subclavian artery rupture.
• We report a case in which the balloon was smaller than the injured subclavian artery lumen.
• We recommend consideration of using smaller balloon diameters to halt extravasation while preserving brain and hand perfusion, and to avoid worsening local subclavian arterial trauma.

## 1 Introduction

Central lines are deployed by the thousands. Indications include rapid volume resuscitation, hemodialysis, parenteral nutrition, and multiple drug/vasopressor therapies. Most frequent approaches are via femoral, internal jugular, and subclavian veins [1]. 30 % of nonimage guided and 18 % of image guided central lines result in failures and/or complications [2]. Arterial injury is reported in 3.7–8 % of cases [3]. The carotid artery is the most frequently injured artery (6–25 % of cases) while the subclavian artery is injured in 0.5–4 % of patients [4].

Complications include arteriovenous fistulas, pseudoaneurysms, massive or tension hemothorax, strokes, and potential airway obstruction from expanding hematomas [1,4]. These complications are potentially exacerbated by larger catheter diameters and longer indwelling periods [5]. One study reports a 47 % complication rate, including stroke and death, when inadvertently entered carotid or subclavian arteries are removed, and hemostasis attempted only by manual compression [3].

Choosing balloon diameter equal to or exceeding arterial diameter is virtually universally advised in order to effect cessation of extravasation [2,3,6]. We report a case in which the balloon was smaller than the injured subclavian artery lumen.

## 2 Case presentation

A 21-year-old male with a history of migraine headaches and iron deficiency anemia was brought to the ER due to gunshot to left thoracoabdomen. Penetrating injury to the left diaphragm, grade IV splenic laceration, right 10th rib fractures and bilateral hemothoraces were discovered by Computed Tomography (CT) and intraoperatively.

**Fig. 1:**
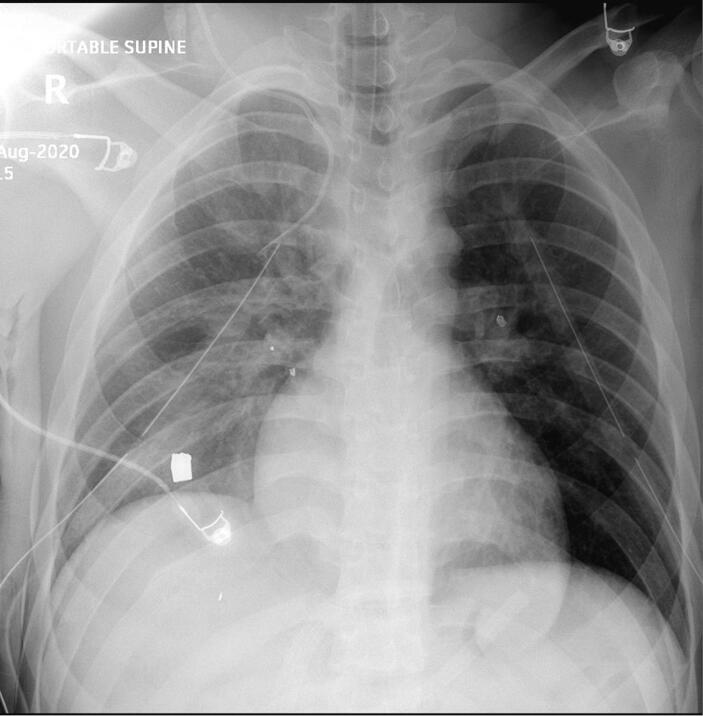
Chest X-Ray reveals malpositioning of the central venous catheter on the right.

As a result, bilateral chest tubes were deployed in the trauma bay and the patient was transferred to the operating room (OR). Right internal jugular puncture was attempted in the OR by deploying a 7F triple lumen catheter. No blood return was encountered and a chest X-ray revealed malpositioning (Fig. 1).

**Fig. 2:**
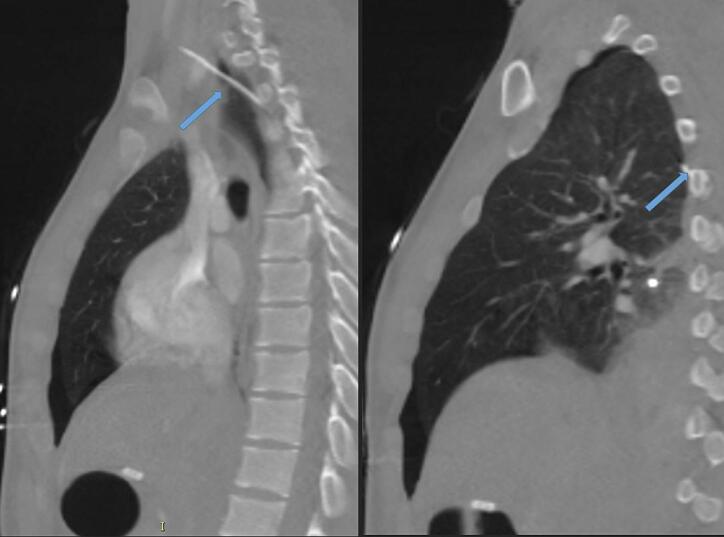
(a) Sagittal view of CT scan revealing puncture through the right subclavian artery (arrow). (b) Sagittal view of CT scan reveals formation of pneumothorax from gun-shot wound.

The catheter was never utilized for infusions. Multiphase CT was obtained postoperatively, which revealed suspicion for puncture through the upper convexity of the right subclavian artery without active extravasation (Fig. 2).

**Fig. 3:**
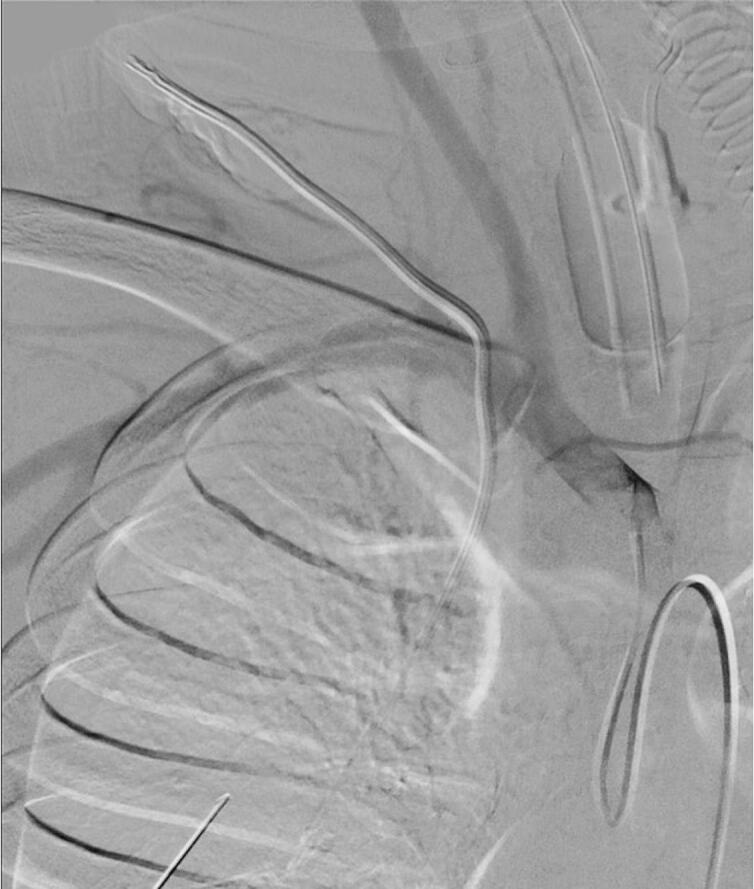
Angiogram showing proximity of carotid and vertebral artery.

Interventional radiology and vascular surgery were consulted. Hence, arteriography was commenced via right femoral puncture. Following exchanges, a sidewinder catheter was utilized to select the origin of the right subclavian artery (Fig. 3). Arteriography again confirmed no extravasations while the malpositioned right central line remained in place.

**Fig. 4:**
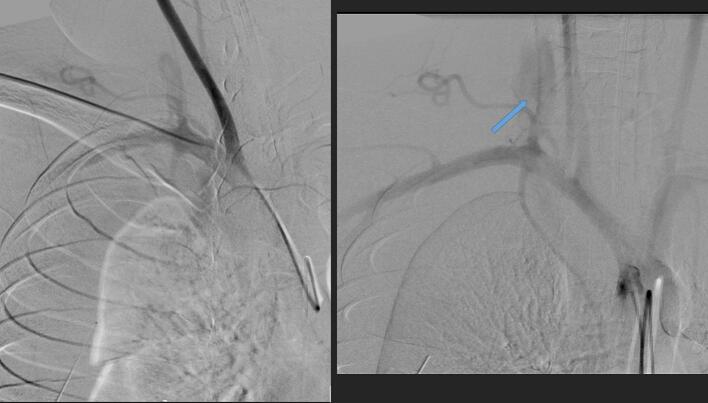
(a) Arteriogram shows enlargement of hemothorax. (b) Arteriogram shows extravasation (arrow) seen after removal of the catheter.

The central line was removed and arteriography was repeated, demonstrating prompt extravasation and rapid enlargement of the right hemothorax (Fig. 4).

**Fig. 5:**
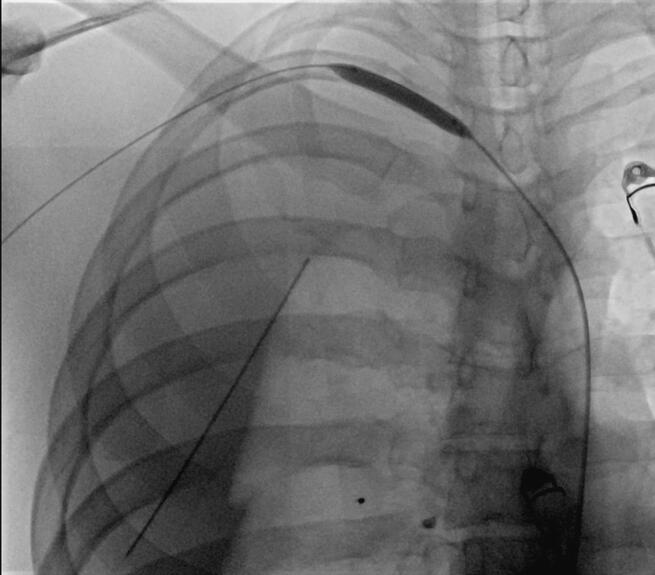
An X-ray shows the use of an undersized balloon used to control extravasation.

Soon thereafter, the systolic blood pressure dropped from 110 mmHg to less than 70 mmHg and the heart rate increased from 112 to 145 beats per minute (BPM). The diagnostic sidewinder catheter was quickly retracted to straighten its preformed loop. An exchange wire was advanced, and the sidewinder catheter was removed. A 7 mm balloon catheter was advanced through the original 5F sheath (Fig. 5).

The balloon was inflated and gently retracted so that its “heel” was situated at the uppermost convexity of the right subclavian artery. These maneuvers required less than 2 min. The patient was rapidly resuscitated with packed red blood cells and crystalloid solutions while the surgical staff evacuated much of the right hemothorax. The systolic blood pressure improved to 110 mm and the heart rate diminished to 110 bpm.

**Fig. 6:**
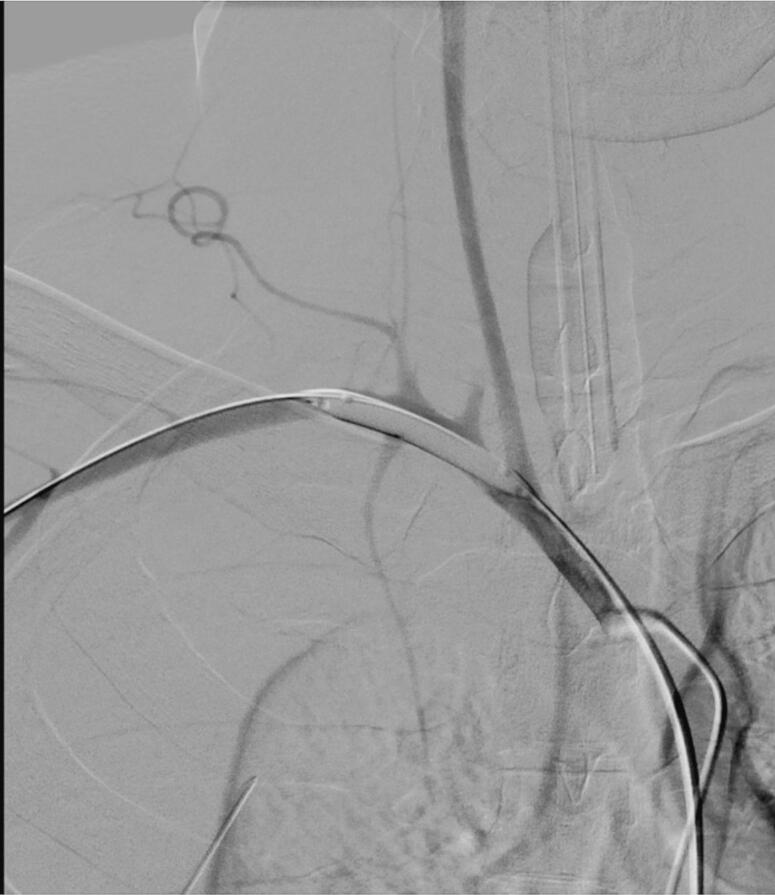
Arteriogram shows an inflated balloon with preserved flow to the carotid, subclavian and axillary arteries with slightly damped flow to the right vertebral artery.

**Fig. 7:**
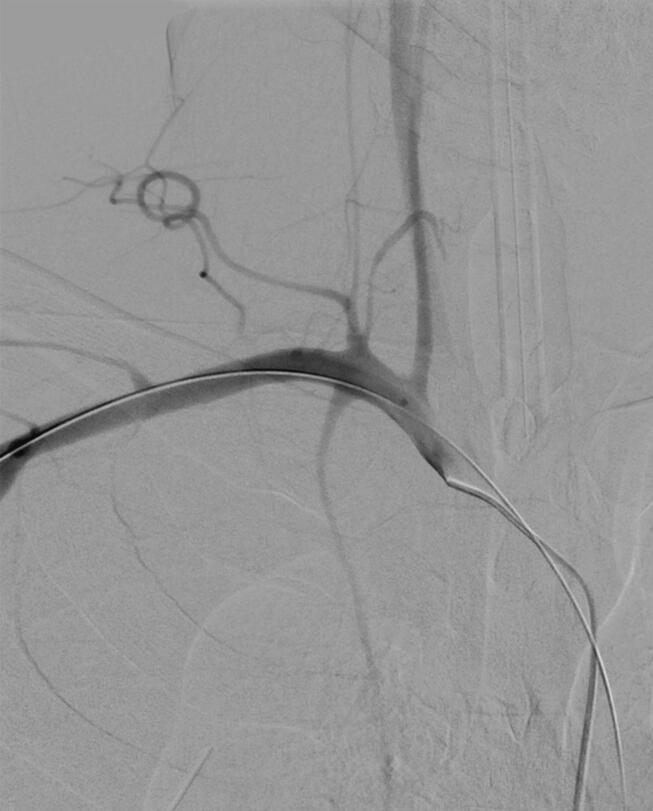
Arteriogram shows balloon removed with the wire left in place revealing excellent flow.

While the balloon was inflated, repeat arteriography was performed following advancement of left coaxial apparatuses via left femoral puncture (Fig. 6). No extravasation was observed. Mild dampening of right vertebral flow was noted; however, rapid right carotid and axillosubclavian arterial flows were maintained. After 25 min, the balloon was deflated but kept in place and arteriography repeated (Fig. 7). No extravasations or local pseudoaneurysms were seen. Moreover, excellent flows were visualized via right axillosubclavian, carotid and vertebral arteries. The balloon catheter was removed over a wire. Arteriography was repeated demonstrating excellent, desired result. Vital signs continued to improve as well. Bilateral femoral coaxial devices were removed and hemostasis achieved at the femoral puncture site using closure devices and manual compressions.

The remaining hospital course was complicated only by leukocytosis with left shift, which was treated with antibiotics and the patient was discharged home 16 days following admission.

The work has been reported in line with the SCARE criteria [7].

## 3 Discussion

Numerous authors advocate that expanded balloon diameters must equal or exceed arterial lumen diameters for balloon occlusion and for stent grafts [2,3,6,8]. We present a balloon occlusion case in which expanded balloon diameter was purposefully smaller than arterial diameter, and in which no additional interventions were necessary. Cutoff of flow to the upper extremity is anticipated when the expanded balloon exceeds subclavian diameter. No clear data exists regarding allowable maximum balloon inflation times at which upper extremity ischemia is anticipated. One study reported balloon inflation time up to 50 min with no ischemic symptoms [3]. Deprivation of carotid or vertebral supply may result in in situ thrombosis and in brain infarction. Carotid balloon test occlusion (BTO) has been associated with reduction of permanent internal carotid artery (ICA) occlusion for 26 % to 13 % [9]. In our case, vertebral flow was temporarily dampened while rapid carotid and axillosubclavian arterial flow was maintained throughout. Therefore, if continued leakage had been demonstrated moments after balloon deflation, we could have simply reinflated our balloon without or with transfer to the OR. We had already agreed not to deploy a stent graft due to proximity of penetration to common carotid origin. Penetration may exceed one small hole, and/or may be laceration(s) rather than hole(s). Balloons when inflated will invariably alter arterial anatomy and orientation, and will potentially exacerbate penetrating injury. These considerations further justify smaller balloon diameters. Several authors advise the use of a long introducer so that all percutaneous interventions are performed via single femoral artery puncture. We opted to perform follow up diagnostic arteriography via a separate femoral artery puncture. Arteriograms through a long introducer and around a recently deflated balloon would have been less robust. The introducer plus deflated balloon catheter may have limited flow to the arm and brain, and might also have adversely influenced subclavian anatomy.

In 1991, Milford et al. reported the first case of successful hemostasis achieved solely unitizing subclavian artery balloon occlusion [10]. A 1987 report describes balloon occlusion to control iliac arterial bleeding without any subsequent open surgical intervention [11]. Milford et al. describe a case in which a 7-French PTA catheter with a 10-mm balloon 4 cm in length was placed distal to the vertebral origin, while overlapping with the catheter's entry site. In contrast, this case involved the use of a 7-mm balloon slightly smaller than the length of the subclavian artery. The use of a balloon occlusion in both case studies helped control the hemorrhage with minimal blood loss, while only temporarily disrupting distal blood flow. Furthermore, if post-surgical procedures were required, the balloon provided proximal control of the artery [9]. To our understanding, only one other case documents the use of a balloon shorter than the vessel diameter. In fact, the balloon used in this case was 6-mm and was used to repair an iatrogenic external iliac artery rupture during a percutaneous transluminal angioplasty [6,11].

Open repair, stent graft procedures, and percutaneous closure devices have also been described. Two studies, one done by Mckinley et al. and another by Degiannis et al. demonstrated failure rates of about 5 % following open repairs. Complications include postoperative stenosis and occlusions [1]. Park et al. claim and demonstrate that endovascular treatment is associated with lower mortality rates than open surgical repair [12].

Injured subclavian artery stent grafts are associated with technical successful rates of 94–100 %, with procedure-related complications between 0 and 22 % [13]. However, stent-grafts are also associated with local thromboembolism and intimal hyperplasia. Patients receiving stent grafts are typically based on prolonged anticoagulation. Prolonged anticoagulation is usually not rendered necessary following balloon occlusion [12]. Moreover, stent grafts are far more expensive [14].

Case reports exist past nine years regarding percutaneous closure devices for similar subclavian injuries. These devices are rapidly gaining popularity; however, they're designed for the femoral artery rather than the (usually softer) subclavian artery. Recent reported complications include complete arterial thrombosis and/or distal embolization with resultant ischemia [1].

## 4 Conclusion

Balloon occlusion offers a less invasive alternative to surgery or stent-graft placement in the treatment of subclavian artery rupture. We recommend consideration of using smaller balloon diameters to halt extravasation while preserving brain and hand perfusion, and to avoid worsening local subclavian arterial trauma.

## Declaration of competing interest

The authors declare that they have no known competing financial interests or personal relationships that could have appeared to influence the work reported in this paper.
